# Grammar Is a System That Characterizes Talk in Interaction

**DOI:** 10.3389/fpsyg.2016.01938

**Published:** 2016-12-22

**Authors:** Jonathan Ginzburg, Massimo Poesio

**Affiliations:** ^1^Université Paris-Diderot and Laboratoire d'Excellence-EFLSorbonne Paris-Cité, Paris, France; ^2^Department of Computer Science, University of EssexWivenhoe, UK

**Keywords:** interaction and the competence/performance distinction, semantics of dialogue, non-sentential utterances, self-repair and other-repair, quotation, gestures and multimodal grammar

## Abstract

Much of contemporary mainstream formal grammar theory is unable to provide analyses for language as it occurs in actual spoken interaction. Its analyses are developed for a cleaned up version of language which omits the disfluencies, non-sentential utterances, gestures, and many other phenomena that are ubiquitous in spoken language. Using evidence from linguistics, conversation analysis, multimodal communication, psychology, language acquisition, and neuroscience, we show these aspects of language use are rule governed in much the same way as phenomena captured by conventional grammars. Furthermore, we argue that over the past few years some of the tools required to provide a precise characterizations of such phenomena have begun to emerge in theoretical and computational linguistics; hence, there is no reason for treating them as “second class citizens” other than pre-theoretical assumptions about what should fall under the purview of grammar. Finally, we suggest that grammar formalisms covering such phenomena would provide a better foundation not just for linguistic analysis of face-to-face interaction, but also for sister disciplines, such as research on spoken dialogue systems and/or psychological work on language acquisition.

## 1. Introduction

What should grammars characterize?

Historically, grammars were developed with written language in mind, and providing analyses for examples from written text was the standard task for grammarians. But following Saussure and the American structuralists, *inter alii*, spoken language became a reputable object of study as well. This trend should have strengthened with the rise of generative grammar, whose avowed aim was characterizing the universals underlying linguistic competence, thus not only in cultures in which written language plays a core role in verbal communication, but also in cultures where only spoken language is used–e.g., tribesmen speaking Pirahã in the Amazon or Arapesh in Papua New Guinea.

And yet in practice contemporary theoretical linguistics is typically not interested or able to provide analyses for the rules governing language as it occurs in actual spoken interaction. Its analyses are developed for a cleaned up version of language [e.g., (1b) for the case of (1a)], which omits the disfluencies, interjections, overlapping turns, non-sentential utterances, and *ad hoc* coinages which are ubiquitous in spoken language, as exemplified in (1)–(3):

(1) a. I'm just really anxious, not anxious, anxious is the wrong word, I'm excited about tomorrow. (Roy Hodgson, England Football manager, The Guardian, 10 October, 2013)
b. I'm excited about tomorrow.

(2) 1. A: and they took a bit of my bone away, also in the process, cos it was so like crck crrck (***ad hoc*** **coinage**)
2. B: what did they put there instead?
3. A: didn't put anything!
4. A: it was a huge, it was a big hole it was (**disfluency**
5. B: what's there now
6. A: huh? (**interjection**)
7. B: what is what's in your mouth now?
8. A: there's nothing, I have like this this (**disfluency**
9. A: this piece of gum that's, that you know erm (**disfluency**)
it's just sort of gummed back together
(From the corpus described in Healey et al. (2015)).

(3) 1. Fri: They still haven't figured out, (.) how they're gonna get to the country: < who's gonna take care of huh m:othah while [they're- y'know 'p in the country. on the weekend.(**disfluency**)
2. Dav: [Mm (0.2 secs)(**non-sentential utterance**)
3. Fri: So: (.) you know, (0.8 secs)
4. Fri: an besides tha[:t,
5. Rub: [You c'n go any[way
6. Dav: [Don - Don git- don [get](**disfluency**)
7. Fri: [they] won t be:
8. Dav: Y know there- there s no- no long explanation is necessary (**disfluency**)
9. Fri: Oh noon no: (**interjection**), (**disfluency**)I'm not- I jus: : uh-wanted: you to know that you can go up anyway. = (**overlapping turns**)
10. Rub: = Yeah:. (0.1 secs)(**non-sentential utterance**)
11. Fri: You know. (0.2 secs)
12. Fri: Because-ah (3.3 secs) (**disfluency**)
13. Rub: They don mind honey they're jus not gonna talk to us ever again. = (**overlapping turns**)
14. Dav: = (laughter) / ri:(h)ight) (**non-sentential utterance**)
(From Schegloff (2001)).

This written language bias (Linell, 2005) characterizes work in most contemporary grammatical formalisms, from the Minimalist Programme (Chomsky, 1995) to Head-driven Phrase Structure Grammar (HPSG) (Pollard and Sag, 1994; Ginzburg and Sag, 2000; Sag et al., 2003) to Lexical Functional Grammar (Bresnan, 1982, 2001) to Categorial Grammar (Moortgat, 1997; Steedman, 2001) to Construction Grammar (Goldberg, 1995; Kay and Fillmore, 1999), though, as we shall see, there has certainly been work in some of these frameworks that very directly engages with spoken language.

For some frameworks the bias is explicitly justified, given continued adherence to Chomsky's competence/performance distinction (Chomsky, 1965) and to a view of grammar as ‘the capacity for unbounded composition of various linguistic objects into complex structures … This approach distinguishes the biological capacity for language from its many possible functions, such as communication or internal thought’ (Hauser et al., 2014). Accordingly, some grammarians attempted to delineate *core* phenomena the grammar needs to account for, in contrast to a periphery (Chomsky, 1981)^1^. However, this strategy seems to have little independent justification (Jackendoff, 2005). Another strategy is to cleanly separate processing within the sentence and discourse–oriented processing: see e.g., Frazier and Clifton (2005). But such a strategy, whatever its merits, is not helpful for dealing with various pervasive within–sentence conversational phenomena such as disfluencies and interjections, exemplified above and discussed below. In other cases, less commitment has been explicitly made as to what empirical phenomena grammars need to account for.

The competence/performance distinction was introduced, in part, as a means of providing a justification for formal grammars, formal systems that abstract away from describing language in an interactive setting. A formal grammar, on this view, is taken to provide a theory of grammaticality: such a theory is tested via subjects' intuitions about the forms of a given language abstracted away from their occurrence in conversation. A theory of performance arises by somehow integrating the formal grammar with a parser/generator and a theory of context. One consequence of this has been to exclude from “competence” certain phenomena which intrinsically involve conversational use (though as we will see in Section 2, this exclusion has not been executed in a principled way). One major claim we make in this paper is that grammars need to aim to analyze all aspects of language use—in other words, we subscribe to the early Chomsky's claim that “The behavior of the speaker, listener, and learner of language constitutes, of course, the actual data for any study of language.” (Chomsky, 1959, p. 56). Just as physics takes responsibility for explaining all physical phenomena without restricting itself to, e.g., frictionless abstractions, and biologists do not put to one side duck billed platypuses or non-kin oriented altruism, grammars cannot pick and choose.

Over the past 40 years important contributions by researchers in conversation analysis, cognitive and social psychology (e.g., Hymes, 1972; Allwood, 1976; Schegloff et al., 1977; Levelt, 1993; Clark, 1996; Pickering and Garrod, 2004; Linell, 2009). have highlighted that competence needs to be stated within a conversationally oriented view of language (see also Ono and Thompson, 1995). While this research has yielded many important insights, some of which are mentioned below, it has for the most part not been formulated within formal frameworks of grammar or cognition. Nor has it developed a precise theory of the structure and dynamics of context in conversation. This has allowed the impression to be conveyed that the various phenomena uncovered in this research cannot or should not be described within theories of grammar similar to those used to describing the more traditional ‘cleaned up’ grammatical phenomena. In Section 3 we provide empirical evidence that the interaction-free notion of grammaticality cannot be maintained: on the one hand, phenomena such as quotation and repair can “save” forms that would be rejected in a non-conversational setting; conversely, cross-turn constructions such as various kinds of non-sentential utterances can be well-formed when adjudged outside a conversational context but their parallelism requirements (e.g., cross-turn case matching) requires their acceptability to be judged relative to a context, or as we will prefer to say, relative to an interaction situation. Phenomena such as these cannot be explained within standard conceptions of grammar, or interaction–free conceptions of grammar, as we will call them; these are therefore intrinsically incomplete. One needs grammars that can encode a view of compositionality wherein meaning emerges by combining information from the interaction situation, speech events, and gestures.

But what are the prospects for a formal grammar to be used to analyze language as it occurs in actual spoken interaction? In the paper we make two further claims. First, we argue in Section 5 that whereas no single “Interaction Grammar” yet exists, recent work in theoretical and computational linguistics has shown that one can develop precise accounts of most of the “conversational phenomena” we discuss of a rigor comparable to those found in typical formal grammars. Second, we show that from this work several fundamental constraints emerge that need to be satisfied by any grammatical framework in which such accounts are formulated. These conditions necessarily change the nature of all the existing major frameworks we are aware of.

The structure of the paper is as follows. In Section 2 we briefly discuss some phenomena whose meaning turns out to be intrinsically interactive and which modern grammatical frameworks have treated, though typically without interfacing with a detailed treatment of context. In Section 3 we present linguistic evidence supporting the contention that interaction–free grammar will not work for spoken language, based on an analysis of a wide variety of ubiquitous constructions. In Section 4, we consider evidence from other disciplines that study language, specifically language acquisition and cognitive neuroscience. We argue that this evidence either supports an interaction-oriented view of grammar or is problematic for interaction–free approaches. Section 5 presents the key theoretical notions and constraints on “interaction grammars” that are beginning to emerge from various theoretical proposals; in particular, it includes sketchy accounts, in different formalisms, of all the phenomena discussed in Section 3. Finally, in Section 6 we briefly discuss the implications of our proposal for linguistics and other behavioral sciences.

## 2. Interactional aspects of communication already accepted as part of grammatical competence

It is worth stressing that in fact modern linguistic theory already accepts that grammatical competence governs some ways of communicating information that are only encountered in interaction, from intonation to gestures, and in particular purely gestural forms of communication such as sign language^2^. In addition, it is generally accepted that at least some form of reference to the Interaction Situation, so-called deictic reference, are governed by grammar.

### 2.1. Intonation

It has long been accepted by modern linguistic theory that (some aspects of) this signal are regulated by sentence level grammar in that they interact with the meaning introduced by words and phrases (see e.g., Jackendoff, 1972; Sgall et al., 1973; Krifka, 1992; Rooth, 1993; Erteschik-Shir, 2007 among many). Crucially, some of the meaning conveyed by intonation seem to be irreducibly interaction oriented—the fall-rise intonation contour [the sequence of tones L(ow)H(igh) in autosegmental theory] associated with theme/ground in English is explicated as, roughly speaking, presupposing a certain issue being under discussion, whereas the nuclear pitch accent associated with focus/rheme (the high tone H) as introducing information new for the addressee (see e.g., Roberts, 1996; Steedman, 2014 for detailed accounts); similarly, the French non-falling contours (a sequence ending with an H^*^) is used when the message conveyed is assumed to involve controversy between speaker and addressee (Beyssade and Marandin, 2007). There is considerable evidence that languages express similar meanings via word order (e.g., Catalan, Vallduví, 1992, Greek, Alexopoulou and Kolliakou, 2002), meaning that word order is also implicated interactionally. There are various attempts to integrate such notions into most modern grammatical frameworks (e.g., HPSG, Engdahl and Vallduví, 1996, LFG, Dalrymple and Mycock, 2011, Minimalism, Zubizarreta, 1998). For the most part, these do not interface with representations of context, but see Steedman (2014) for such an account with Combinatory Categorial Grammar and (Vallduví, 2016) for a detailed account of all notions of information structure cast in terms of dialogical context.

### 2.2. Deixis

One type of reference to the Interaction Situation that is generally accepted to be governed by grammar is the information coming from **pointing**. In the account of demonstratives of Kaplan (1978), for instance, every demonstrative *d* is accompanied by a **demonstration** δ—e.g., a pointing gesture–and the grammar provides a semantics for *d*[δ] jointly: specifically, *d*[δ] is a directly referential term that designates the **demonstratum** of δ in context *c*. This account has been widely adopted in modern formal semantics. But the idea that the role of the Interaction Situation in the semantics of demonstratives could be entirely abstracted away through the notion of demonstration is open to significant challenge, as we discuss in Section 3.5.

### 2.3. Gestures

There is considerable evidence that in face-to-face conversation, verbal information is integrated with information from a variety of gestures in addition to pointing (Kendon, 1980; McNeill, 1992; Bavelas and Chovil, 2000; Kendon, 2004). Kendon (2004) charts the fall and rise in the scientific and scholarly status of gestures:^3^ gestures were seen in traditional Rhetoric as a key component of human utterance and public performance. However, gestures lost their status sometime during the nineteenth century—in part because of a shift toward a more controlled style of public delivery in which gestures played less of a role, in part because the printed word came to be seen as the truest form of language expression. This decline in status of gestures was paralleled by a reduced interest in this form of expression in linguistics. Linguists came to question the extent to which the contribution of gestures ought to be considered part of grammar, arguing instead that the role of gestures is purely depictive or pantomimic. In the last 30 years, however, thanks also to technological advances in recording and analyzing videos that have enabled extensive and detailed empirical investigations, gestures have come to be recognized again as a key component of human utterance.

Studies of the relation of gesture and speech using such audio-visual methodology have shown the two activities to be so intimately correlated that they appear to be governed by a single process, as emphasized by the pioneering work of Kendon and McNeill in particular. Recent research e.g., in the ToGoG project has provided evidence that a number of gestures have undergone a process of grammaticalization (Bressem and Ladewig, 2011; Schoonjans, 2013). There is also psychological evidence that such information is immediately integrated (e.g., Ozyurek et al., 2007). It has thus become clear that both gesture and speech make essential contributions to referential meaning, so that one form of expression cannot be considered as primary (Kendon, 2004). One example is **head-shaking** and other gestures used to express negation (Kendon, 2002; González-Fuente et al., 2015). A formal treatment of gestural negation and its grammatical role–in particular, its scope–has been provided by, e.g., Harrison (2010). More generally, recent years have witnessed the development of so-called **multimodal grammars**, which provide an integrated account of both the spoken and the gestural aspect of human utterance (Johnston et al., 1997; Lascarides and Stone, 2009; Poesio and Rieser, 2009; Alahverdzhieva and Lascarides, 2010; Fricke, 2013).

### 2.4. Sign language

Virtually all theoretical linguists view **sign language** as being governed by the same kind of grammar that governs verbal forms of communication (Newport and Supalla, 1999). Accounts of, e.g., the grammar of pronominal anaphora, or the tense system of several sign languages have been proposed that utilize the same ingredients of standard generative grammar (see e.g., Zucchi, 2012).

Like the accounts of the grammatical role of gestures discussed above, such accounts abstract away from references to the Interaction Situation. But much the same issues arise with such abstraction as with the abstraction proposed for the role of pointing gestures in deixis. Indeed, the exact same issues arise for the proposed accounts of anaphoric reference in sign language.

Anaphoric pronouns are usually expressed in sign language by pointing to the spatial locations where the antecedents have been signed. For example, while in English sentence (4) below (Lillo-Martin and Klima, 1991) the relation the pronouns *he* and *him* bear to their antecedents is not overtly marked and needs to be inferred from extra-linguistic clues, in American Sign Language (ASL) the corresponding sentence *is* disambiguated by the **loci** of the pronouns: the locations in space to which the index finger points. If the index points to the location where the sign JOHN was signed, then JOHN is the antecedent of the pronoun, while if the index points to the location where the sign BILL was signed, BILL is the antecedent of the pronoun.

(4) John called Bill a Republican and then he insulted him.

Clearly, the same questions raised with respect to pointing apply to the case of loci identification.

### 2.5. Beyond

In the rest of the paper we argue that there is no principled dividing line between phenomena such as intonation and deixis, widely accepted as falling within the purview of (competence) grammar, and the phenomena we review in Section 3. Given the need to accommodate the former within grammar, this entails a similar conclusion for the latter. This, in turn, requires a view of grammar embedded in interaction, a move which will also lead to a more principled account of ‘information structure’ phenomena.

## 3. Much of our grammatical competence concerns language use in interaction: linguistic evidence

In this section we demonstrate that many pervasive aspects of spoken and written language use are subject to grammatical constraints that cannot be described in interaction-free terms. They show that grammaticality has to be relativized to interaction situations. The phenomena we discuss fall under five broad categories:

- **Grammatical constraints across conversational turns**: parallelism constraints on multiple linguistic levels whose scope ranges across participant turns.
- **Interaction Situation reference**: the existence of systematic, conventionalized dependencies that make explicit, unavoidable reference to the interaction situation.
- **Online repair**: repair phenomena that take place while the utterance is in progress and lead to non-monotonic effects in structure and content construction.
- **Genre dependence**: the impossibility of maintaining a global grammar.
- **Speech-gesture integration**: the need to integrate speech and gesture in content construction.

### 3.1. Grammatical constraints across conversational turns

#### 3.1.1. Greeting

In a wide variety of languages there exist words and phrases whose conventional meaning requires making non-eliminable reference to the existence of a conversation, indeed to the fine structure of a conversation. Greeting words like English ‘hi,’ ‘hello,’^4^ ‘good morning’ must occur conversation–initially or as responses to an immediately prior greeting by another conversationalist. And many languages have more fine grained systems: e.g., Syrian and Lebanese Arabic, where ‘sabaḥ elxeyr,’ ‘marḥaba,’ and ‘bonjour’ occur conversation–initially, whereas ‘sabaḥ elnur,’ ‘marḥabteyn,’ and ‘bonjoureyn’ can only be used as responses to these greetings, respectively (Ferguson, 1967):

(5)  a.  (#)  A:  saba$\underset{˙}{\text{h}}$     elxeyr      B:  saba$\underset{˙}{\text{h}}$      elnur.
           (#)  A:  morning  def-good  B:  morning  def-light
           ‘ A: Good morning B: Good morning’
      b.  (#)  A:  mar$\underset{˙}{\text{h}}$aba  B:  mar$\underset{˙}{\text{h}}$abteyn.
           (#)  A:  hello        B:  hello-dual
           ‘ A: Hello B: Hello’
      c.  (#)  A:  Bonjour  B:  Bonjoureyn.
           (#)  A:  hello       B:  hello-dual
           ‘ A: Hello B: Hello’.

These facts, which require direct reference to conversational structure, need to be registered in some way in the lexical entries of such words. Thus, very similar argumentation to that used by syntacticians to motivate various notions of (intra-sentential) syntactic dependence e.g., cliticisation and complementation could be used to motivate the need for a mechanism that can capture the fact that words like ‘sabaḥ elnur’ and ‘marḥabteyn’ can only be used as responses to greetings.

#### 3.1.2. Parting

By the same token, a wide variety of languages have words and phrases whose conventional meaning involves **parting**. Parting is more complex than greeting—it involves making the judgment that a non-negligible amount of interaction has taken place (Ginzburg, 2012). As with greetings, there exist languages where the parting expression has presuppositions about the form of a preceding parting phrase: in Syrian Arabic, for instance, ‘Allah ya'afik’ requires as preceding utterance the parting phrase ‘ya'atik el'afiye.’(Ferguson, 1967)^5^. This indicates that such form–oriented cross-turn presuppositions apply to multi word expressions as well:^6^

(6)       (#)  A:  ya’atik              el’afiye      B:  Allah  ya’afik.
           (#)  A:  give-3rd-sg-fut  def-health  B:  God  healthify-
                 3rd-sg-fut
           ‘ A: [God] give you health B: God healthify-you’.

#### 3.1.3. Non-sentential utterances

Greetings and partings are just two examples of **non-sentential utterances**: utterances lacking an overt predicate [see examples (1)–(3) above and elsewhere]. Such utterances are ubiquitous in conversations: de Weijer (2001) provides figures of 40, 31, and 30% respectively for the percentage of **one word utterances** in the speech exchanged between adults and infant, adult and toddler, and among adults in a single Dutch speaking family consisting of 2 adults, 1 toddler and 1 baby across 2 months.

Non-sentential utterances are not a motley crowd; recent studies have shown that they can be reliably classified into a small number of categories, revolving around the commonality in semantic resolution process (see e.g., Fernández and Ginzburg, 2002; Schlangen, 2003). And yet, clearly a non-sentential utterance has little content outside a conversational context. (7) illustrates that this same form can receive highly diverse contents from a wide range of sources: a previously uttered question, a question implicit in a particular domain, and as a correction:

(7) a. B: Four croissants.
b. (Context: A: What did you buy in the bakery?) Content: I bought four croissants in the bakery.
c. (Context: A smiles at B, who has become the next customer to be served at the bakery.) Content: I would like to buy four croissants.
d. (Context: A: Dad bought four crescents.) Content: You mean that Dad bought four croissants.

Thus, the competence in producing and understanding such utterances involves the context in an unavoidable way, including, as exemplified in (7b), how utterances fit in with social interaction. Conversely, matters of form can themselves, in the general case, require reference to the context. It was already pointed out by Lakoff (1971) and Morgan (1973)—though subsequently largely forgotten–that non-sentential utterances provide evidence that grammaticality cannot be adjudged context independently, i.e., simply by considering the morphosyntactic properties of a string. (8a,b) involve two virtually synonymous questions that lead to distinct contexts. (8a) is compatible with a possessive NP as response, but not with a nominative NP, whereas in (8b) this pattern is reversed.

(8) a. A: Whose book did you borrow? B: Jo's. /# Jo
b. A: Who owns the book you borrowed? B: # Jo's. / Jo. / It's Jo's.

Viewed from the perspective of the non-sentential utterance, this pattern suggests that the non-sentential utterance ‘Jo's’ has a presupposition that, to the extent its antecedent derives from a linguistic utterance, it must bear genitive case. Cross–turn dependencies of this kind are common among various types of non-sentential utterances, across a wide range of languages (Ross, 1969; Merchant, 2001; Sag and Nykiel, 2011; Ginzburg, 2012). What bears emphasizing is that such dependencies can stretch across many turns, particularly in multi-party dialogue, thereby reinforcing the need for this information to be in long-medium term representation of context: Ginzburg and Fernández (2005) found that in the British National Corpus (BNC) over 44% of short answers have more than distance 1, and over 24% have distance 4 or more, as in the constructed example in (9):

(9) a. A(1): Who is coming to the barbecue?
B(2): the barbecue on Sunday?
A(3): the 29th yes
B(4): Sunday is the 28th.
A(5): Oh right, yes the 28th.
B(6): The one Sam's organizing?
A(7): Yes.
B(8): Will it be on even if it snows?
A(9): Sam hasn't said anything.
B(10): Right. Anyway, I'd guess Sue and Pat for sure, maybe Alex too.

### 3.2. References to the interaction situation

In this section we discuss a number of constructions, many of which utterly ubiquitous, that make reference to the ongoing interaction situation.

#### 3.2.1. References to events in the interaction situation

Deictic reference to objects simultaneous with pointing, of the type already discussed, is not the only form of reference to aspects of the interaction situation. There are a number of other expressions, particularly in spoken written language use, whose referent can only be described by in terms of events in the interaction situation.

Most current theories of discourse structure–e.g., SDRT (Asher and Lascarides, 2003)—assume that connectives involve some sort of implicit reference to illocutionary events. And indeed explicit references to illocutionary acts are also possible, as in the following example, where demonstrative *that* in the second utterance is a reference to the promise.

(10) a. A: John, I promise I will help you with your homework.
b. B: That was silly, as you won't have any time.

But locutionary events can be referred to as well. Webber (1991), for instance, discusses examples like (11), in which demonstrative *that* and pronoun *it* refer to the locutionary act performed with the first utterance.

(11) a. A: The combination is 1-2-3-4.
b. B: Could you repeat that? I didn't hear it.

#### 3.2.2. Clarification requests

Plato was already at least implicitly aware of the fact that language enables one to explicitly address communicative aspects of an utterance: the Socratic dialogues are replete with examples of utterances whose primary function is to serve as **clarification requests** (CRs), in other words to indicate that some aspect of a prior utterance, typically its meaning, is unclear:

(12) a. Her. Yes; but what do you say of the other name?
  Soc. Athene?
  Her. Yes.
b. Soc. There is no difficulty in explaining the other appellation of Athene.
  Her. What other appellation?
  Soc. We call her Pallas. (From Cratylus, http://en.wikisource.org/wiki/Cratylus).

CRs can take many forms, as illustrated in Table 1, a taxonomy based on CRs occurring in the British National Corpus.

**Table 1:** **A taxonomy for clarification requests, Table 1 from Purver (2006)**.

**Category name**	**Example**
Example context	A: Did Bo leave?
Wot	B: Eh? / What? / Pardon?
Explicit	B: Did you say ‘Bo’ /
	What do you mean ‘leave’?
Literal reprise	B: Did BO leave?
	Did Bo LEAVE?
Wh-substituted Reprise	B: Did WHO leave?
	Did Bo WHAT?
Reprise sluice	B: Who? / What? / Where?
Reprise Fragments	B: Bo? / Leave?
Gap	B: Did Bo …?
Filler	A: Did Bo …B: Win?

Providing explicit formal analyses of just about any of these classes is a formidable challenge for most existing formal grammatical frameworks. We highlight just a few of the most significant issues.

The first point to note is that for a number of these forms the *sole* analysis is as clarification requests: this applies to the classes **Wh-substituted Reprise** and **Gap**. The meanings of such forms cannot be analyzed in interaction–free grammar.

A second point relates to cross-turn parallelism. Ginzburg and Cooper (2004) and Ginzburg (2012) argue in detail that **reprise fragments** have two main classes of uses, one to request confirmation about the content of a previous sub-utterance, the other to find out about the intended content of a previous sub-utterance. Both uses have strong parallelism requirements. The former requires identity of morphosyntactic category between source and target, as illustrated in (13a,b). The latter requires segmental identity between source and target, as exemplified in (13c). Parallelism of the latter kind seems needed also for the **Gap** class of CRs:

(13) a. A: Did she hit him? B: #he/ him
b. A: Was she biking? B: biking/cycling/#biked?
c. A: Did Bo leave? B: Bo? (Intended content reading: **Who are you referring to?** or **Who do you mean?**) Alternative reprise: B: Max? lacks intended content reading; can only mean: **Are you referring to Max?**).

A final point concerning CRs involves anaphora: CRs typically involve anaphoric reference to utterance tokens. This is, in fact, a more general requirement concerning quotative acts in dialogue, to which we return below.

(14) a. A: Max is leaving. B: leaving? (=What does ‘leaving’ mean in the A's sub-utterance, NOT in general.)
b. A: We're fed up. B: Who is we? (=*Who is we* **in the sub-utterance needing clarification**).

#### 3.2.3. Order-dependent expressions

So-called **‘Metalinguistic’ expressions** are expressions whose interpretation depends on the way other utterances have been pronounced, or on the order in which other expressions have been uttered. We will concentrate here on metalinguistic expressions whose interpretation is affected by the order in which other expressions are uttered or occur in a text, such as *the former/the latter, vice versa, respectively*, and *the following* (McCawley, 1970; Kay, 1989; Corblin, 1999; Yamauchi, 2006). The uses of these expressions we are interested in are illustrated in (15a) and (15b):

(15) a. Bob and John were at the meeting. *The former* brought his wife with him. (Quirk/Greenbaum)
b. I think actors can teach dancers a lot, and *vice versa*. (From the British National Corpus.)

*Former* in (15a) has a different meaning from the meaning it has in expressions like *George Bush, the former president of the US*. Intuitively, the semantics of the definite description *the former* in examples like (15a) can be specified informally as follows: the definite description denotes that element of a familiar set of individuals that is denoted by the first NP used to introduce an element of that set. In other words, these definite descriptions behave like the definite description *the yellow one* in *Bill Singer bought two shirts. The yellow one had red buttons*, except that the identifying property is metalinguistic: it refers to the order of elements in the text.

Regarding *vice versa*, it seems reasonable to assume that the use of *vice versa* exemplified by (15b) denotes the proposition which is the content of the statement obtained by exchanging two elements of a previous statement; i.e., that *vice versa* in (15b) denotes the proposition that is the content of the statement *dancers can teach actors a lot* obtained by reverting the order of two sub-utterances of (15b), the utterance of *dancers* and the utterance of *actors* (Culicover and Jackendoff, 2012).

How can we make this informal semantics of order-dependent expressions more precise? One might think that the semantics of *the former*, at least, could be specified within a framework like Heim's File Change Semantics (Heim, 1982), by assuming that an ordering exists on the set of file cards posited to underlie reference resolution in the theory. More specifically, one could propose that the sense of *former* under discussion is a predicate that is satisfied by the element of a set iff the file card associated with that element precedes the file cards associated with the other elements of the set. And indeed, a proposal of this type was made in Corblin and Laborde (2001). Corblin and Laborde propose that the common ground consists of two parts: a part containing information about the propositional content of utterances, and a part so-called *mentionelle* containing information about the mentions of file cards. Two observations can be made about this approach. First, that the information *mentionelle* is in effect information about a subset of the utterances in the Interaction Situation–namely, the utterances of NPs. Second, that in order to account for the entire range of order-dependent expressions, more is needed. This is because *vice versa*, in particular, can refer to the order of virtually any sentence constituents, not just noun phrases. In the famous Dorothy Parker joke *I'm too fucking busy, and vice versa*, for example, the constituents that get ‘switched’ are an adjective and an intensifier, and the switching affects their syntactic interpretation as well as their meaning. This suggests that in the general case a more general form of metalinguistic reference can be used, involving references to various types of utterances of syntactic constituents in the Interaction Situation.

#### 3.2.4. Turn taking

As first pointed out in the seminal paper by Sacks et al. (1974), interlocutors manage turn allocation remarkably well. This has often been summarized as **no-gap-no-overlap**, though Heldner and Edlund (2010), based on a study of corpora in Dutch, Swedish, and English, conclude that sizable departures from no-gap-no-overlap occur frequently, while cases with neither gap nor overlap are very rare: gaps with a duration above the threshold for detection of silences represent more than 40% of all between-speaker intervals in their material, whereas overlaps represent about 40% of all between-speaker intervals. Levinson and Torreira (2015) dispute certain of the conclusions of Heldner and Edlund (2010), specifically the doubts the latter cast on the Sacks et al model, and emphasize the challenges turn taking poses for existing psycholinguistic models of language processing. How turn taking is achieved clearly involves a complex interaction of cues, initially morphosyntactic ones, these later interacting with intonational ones (Levinson and Torreira, 2015) and is also strongly conditioned by content—it is, for instance, infelicitous to respond gaplessly to a complex question (Heldner and Edlund, 2010). However, regardless of the precise division of labor, it is clear that some aspects of turn taking are grammaticized. Thus, the collocation ‘go ahead’ is used to cede the turn, typically when there has been overlap, as exemplified in (16a). (McCarthy and O'Keeffe, 2003) show that turn management is one of the important uses of vocatives particularly in multi-party dialogue, as illustrated in (16b,c). In such cases the fact that the turn has been assigned to the person addressed is, arguably, part of its conventional meaning.

(16) a. A:yeah that's that's kind of strange [laughter] that we got the same call B:[laughter];yeah uh uh A:it to whi[ch]- oh B: oh i'm sorry go ahead A: no that's okay (Switchboard corpus Godfrey et al. (1992), 2053:62)
b. A: I should have some change. B: I owe you too don't I, Jodie. C: Yes you do. (Example (14) from McCarthy and O'Keeffe (2003))
c. [A caller (C), a nurse, is talking about the special vocation of nurses and doctors. Monica Hall (B), a nurse wrongly accused of murdering a colleague in Saudi Arabia, is already on the line] C: … I'm sure Monica would agree with me on this its a sort of kind of spiritual relationship. You're all fighting for the one thing and that one thing is to preserve and improve life for people. A: Monica? B: Yes I I would agree there M= Mari is it? (Example (28) from McCarthy and O'Keeffe (2003)).

Representing turn management in a collocation such as ‘go ahead’ or in a turn assigning use of a vocative requires means of stating within the grammar information such as ‘referent of this NP is hereby offered the next turn.’

### 3.3. Online self-repair/own communication management

As we saw in examples (1)–(3), conversations are littered with disfluencies, or as we would prefer to describe it, conversationalists continually utilize **own communication management** (OCM) devices to correct or modify their utterances or to gain extra time when facing lexical access or utterance planning problems^7^. Although own communication management is viewed as a performance phenomenon in most formal grammatical treatments—a view explicitly rejected by psycholinguists e.g., Levelt (1983), Clark and FoxTree (2002), Ferreira (2005), the unity it displays with Other Communication Management (clarification questions and other–corrections) was noted already in the seminal paper (Schegloff et al., 1977).

Probably the main substantive reason for pushing OCMs to the performance wastebasket is the assumption that they constitute **noise**. But in fact, far from constituting meaningless noise, OCMs participate in semantic and pragmatic processes such as anaphora, conversational implicature, and discourse particles, as illustrated in (17–19). In (17), the semantic process is dependent on the **reparandum** (the phrase to be repaired) as the antecedent:

(17) a. Peter was, well, he was fired. (Example from Heeman and Allen (1999); anaphor refers to material in reparandum)
b. A: Because I, any, anyone, any friend, anyone, I give my number to is welcome to call me (Example from the Switchboard corpus (Godfrey et al., 1992); implicature based on contrast between repair and reprandum: ‘It’s not just her friends that are welcome to call her when A gives them her number').

Moreover, utterances containing disfluencies constitute a subclass of the antecedents for various linguistic expressions, including ‘no’ and ‘or’:

(18) a. From yellow down to brown - no - that's red. (From Levelt (1983))
b. Over the gree-, no I'm wrong, left of the green disk…
c. A: Is Bill coming? B: No, Mary is.
d. [A opens the freezer to discover a smashed beer bottle] A: No! (‘I do not want *this* (the beer bottle smashing) to happen’).

(19) a. We go straight on, or– we enter via red, then go straight on to green. (From Levelt (1983))
b. The design of or– the point of putting two sensors on each side. (From Besser and Alexandersson (2007)).

Non-disfluent speech is analogous to frictionless motion. Some of the time it is useful to ignore the effects of friction, but the theory of motion is required to explicate the existence and quantitative effects of friction. Whereas it seems plausible that not all disfluencies are consciously produced *by the speaker*, for the addressee they always form part of the verbal string as perceived which needs to be parsed and interpreted.

Moreover, OCMs illustrate the primacy—or at least equal footing—of the speech event over grammatical form:^8^ as Levelt (1983) has observed, speakers will stop in ‘mid word’ when detecting error, as exemplified in (20a,b)—in the latter apparently the speaker replaces the beginning of the verb ‘instruct’ with the less specific verb ‘do’; moreover, speakers can stop in mid-utterance if the intended meaning seems to have been communicated—in (20c) D produces a clause headed by the complementizer ‘whether’, omitting a subordinating predicate (e.g., ‘is unclear/unlikely’ etc) and both he and A laugh together about the mutually communicated content:

(20) a. We can go straight on to the ye–, to the orange node. Levelt (1983)
b. Bee: y'know they(d) they do b- t!.hhhh they try even harder than a- y'know a regular instructor. /Ava: Right. / Bee: hhhh to uh insr yknow do the class and everything. (example (18) from Fox et al. (2010))
c. D: lots of secretaries do that, but it's such a waste of time, but on the other hand you do meet / A : yes / D: secretaries. Whether you want to meet secretaries … A,D: (laugh) (From the London Lund corpus, Svartvik and Quirk (1980)).

Indeed, OCM utterances display an important characteristic of grammatical processes, namely cross-linguistic variation. This has been documented in some detail in comparative work between morphosyntactic aspects of repair on a wide range of languages by Fox et al. (e.g., Fox et al., 1996; Wouk et al., 2009; Fox et al., 2010). For phonetic analysis of cross-linguistic variation see Candea et al. (2005), who compare fillers in Arabic, Mandarin Chinese, French, German, Italian, European Portuguese, American English and Latin American Spanish. They demonstrate that language-specific features can be observed in the segmental structure of the fillers. French, for example, prefers a vocalic segment as filler realization, whereas English prefers vowels followed occasionally by a nasal coda consonant [m]. Moreover, while for some languages the vocalic support of the fillers might be a segment exterior to the vocalic system of the language, in all the eight languages the fillers' vocalic support involves at least one of the vowels of their vocalic system.

There is some variation in how hesitation is typically expressed in various languages, as exemplified in (21). Indeed, some languages, e.g., Chinese and Finnish exemplified in (21c,d), use demonstratives for this role:

(21) a. ‘uh’ ‘um’ (English) (Clark and FoxTree, 2002)
b. ‘euh’ … (French): tu sais c'était un peu euh l'ambiance santa-Barbar- euh (De Fornel and Marandin (1996), example (1a))
c. Chinese: ‘en’, ‘nage …’ repeated *ad libitum* (literally ‘that …’), ‘zhege’ (literally ‘this’) (Zhao and Jurafsky, 2005)
d. Finnish: ‘tuota noin’ ('that so'), , or 'tuota …' repeated *ad libitum* ('that …')^9^.

Clark and FoxTree (2002), following an earlier proposal by James (1972) and based on data from the London Lund corpus, claim that the choice of ‘um’ vs. ‘uh’ reflects an explicit choice by the speaker—the former selected when the speaker faces a relatively significant difficulty which will lead to a longer wait for the resumption of the utterance; for dissent against this claim see e.g., O'Connell and Kowal (2005) and Corley and Stewart (2008) Recently, Tian et al. (2016) demonstrate significant preference for ‘um’ v. ‘uh’ among speakers of British English before self addressed questions (e.g., ‘What do they call it?’ ‘what's her name?’)—a clear signal of major difficulty, but no significant difference among speakers of American English (data from Switchboard); they also demonstrate marked preference for certain hesitation markers in Japanese and Chinese on the basis of distinct syntactic contexts. Wieling et al. (2016) demonstrate significant differences in the choice of ‘um’ vs. ‘uh’ (and their cross-linguistic variants) both between male/female and younger/older speakers in four Germanic languages (Dutch, English, German, and Norwegian). This emergent body of work supports the claim that hesitation markers are words the choice between which reflects explicit speaker intention.

Additional reasoning supporting the need for incorporating disfluency markers in the grammar are the following considerations: a child acquiring English needs to discover that ‘no’ can be used in a self-correction, but, for instance, the closely related word ‘nope’ cannot. A trilingual acquiring English, German, and French will need to learn that French ‘enfin’ can be used in a self-correction, whereas English ‘finally’ and German ‘schließlich,’ which are often interchangeable with ‘enfin,’ cannot be so used:

(22)    Quand ma belle      mère’   **enfin** quand ma femme apelle
           When my in-law     mother  **enfin** when  my wife     calls
           ‘When my mother in–law I mean when my wife calls’
           (De Fornel and Marandin, 1996, example (2a)).

Conversely, Ginzburg et al. (2014) suggest that OCMs are also involved in grammatical universals. Based on evidence from 7 languages, they postulate the following:

(23) If NEG is a language's word that can be used as a negation and in cross-turn correction, then NEG can also be used as an editing phrase in backward-looking disfluencies.

### 3.4. Why there cannot be a global grammar: evidence from quotation

The phenomenon of direct quotation perhaps epitomizes the point of the paper: it is ubiquitous, it is subject to grammatical constraints, but features in few formal grammars (for some recent formal treatments see Geurts and Maier, 2005; Potts, 2007; Bonami and Godard, 2008, but these do not form part of a large scale grammar). In a way this is not surprising since, as argued by Ginzburg and Cooper (2014), quotation is a challenge for any grammar *G*: for any string *e* deemed ungrammatical by *G*, one can produce via quotation a well formed string that includes *e*, hence undermining *G*. Thus, we can quote something that is ungrammatical in our own language as in (24a) or something that is in a different language to the one we are speaking (24b), sounds made by inanimate objects (24c), or the thoughts of non-humans (24d).

(24) a. Damien, who's only four years old, said ‘I go’ed to Grandma's'
b. Pelle, whose native language is Swedish, said ‘Jag har varit hos mormor’ (meaning “I've been at Grandma's”)
c. The blender went ‘plplplpl’
d. [An article about an orphaned walrus arriving in a new zoo:] During [the orphan walrus's] first look at a walrus, he was like, ‘What’s that?' (New York Times, 22/01/2014).

Given the diversity of quotable stuff, one might very well think it beyond the remit of somebody writing a formal grammar of English to characterize everything that can occur between quotation marks in sentences like those in (24). Such a strategy is, however, not tenable, for reasons mostly pointed out already by Partee (1973), who provides a variety of examples where the form or the content of the quotation is referred to from outside the quotation as in (25).

(25) a. ‘I talk better English than the both of youse!’ shouted Charles, thereby convincing me that he didn't.
b. The sign says ‘George Washington slept here’, but I don't believe he really did.
c. What he actually said was, ‘It’s clear that you've given this problem a great deal of thought,' but he meant quite the opposite.

And indeed there is substantial evidence that quotation is subject to general grammatical principles governing word order, ability to be embedded and pseudo-clefted, and semantic selection (Postal, 2004; Bonami and Godard, 2008). For instance, there are words in numerous languages that require direct quotation as their complements. In English the marker *like* and the verb *go* have a certain usage which requires a direct quotation as in (26a) and (26b) and does not allow an indirect quotation, as exemplified in (26c) and (26d).

(26) I asked her if she wanted to read my paper
a. and she was like “Are you crazy?”
b. and she went “Yuck!”
c. ^*^ and she was like whether I was crazy
d. ^*^ and she went that she didn't, in no uncertain terms
(examples from Ginzburg and Cooper (2014)).

Such constructions exist in many, if not all, languages although they tend to be restricted to an informal spoken register, see e.g., French *faire, genre*, German *quasi*, Italian *tipo*, and Swedish *typ/ba*. Moreover, all natural languages seem to have direct quotation of some kind. Children use direct quotation from their earliest utterances (Ginzburg and Moradlou, 2013). Given the ubiquity of quotation in natural language, linguists need to explicate the mechanisms it employs. Indeed, one is obligated to do so in a way that offers an answer to the question: why, rather than being a heterodox linguistic process, is in fact quotation so straightforward? We will suggest one such answer below. Whatever one proposes, it seems clear that direct quotation is a grammatical construction where reference is made to an interaction act, constrained via a similarity relation that needs to hold between the quoted material and the original act; Ginzburg and Cooper (2014) argue that the nature of the similarity relation is a contextual parameter of this construction, as is local grammar—the system of rules used to classify the original act. Most crucially, it forces the grammar to be an intrinsically *open* system (Harris, 1979; Postal, 2004).

### 3.5. Pointing, gestures and the interaction situation

The view of the role of pointing and other gestures in communication, as discussed in Section 2, that essentially abstracts away from the Interaction Situation, has been challenged in a number of ways.

#### 3.5.1. Pointing

Extensive empirical work by Lücking, Pfeiffer, Rieser and colleagues at the University of Bielefeld (Kranstedt et al., 2006; Lücking et al., 2015) using highly sophisticated recording and visualization equipment has demonstrated that pointing gestures seldom if ever function as unique identifiers of a demonstratum, as proposed by Kaplan (1978). In all but the simplest situations, the identification of the demonstratum among the objects in the **pointing cone** identified by a pointing gesture is a complex reasoning process involving consideration of a number of additional aspects of the Interaction Situation.

Beyond this, Clark (2003) showed that pointing is neither the only nor the prototypical way to carry out a demonstration. For instance, a customer can felicitously demonstrate to the teller in a supermarker the referents of a demonstrative like *These two things over here* by **placing** the two objects on the counter rather than merely pointing at them.

#### 3.5.2. Interactional role of other gestures

Kendon (2004) distinguishes between two types of gestures: gestures that contribute to what he calls the ‘referential meaning’ of the utterance (discussed in Chapters 9–11) and ‘pragmatic’ gestures (discussed in Chapters 12 and 13). Among the latter, there are several whose function is to manage aspects of the Interaction Situation. These include gestures whose function is to indicate to whom a current utterance is addressed, and several gestures that play a role in turn-taking: for instance, indicating that the speaker is holding the floor, or raising a hand to request a turn, or pointing to indicate the next to hold the floor.

(27a) exemplifies a wordless exchange mediated solely by display and gesture, which corresponds to a question/answer pair, as in either (27b), where the question is implicit and the answer is a non-sentential utterance, or (27c), where the question is explicit and the answer is a non-sentential utterance. This indicates the need for a mechanism that unifies all three cases, given the intuitive synonymy.

(27) a. Owner: (displays three fresh fish on a platter) Clark: (points at one of them) (From Clark (2012): example (32))
b. Owner: (displays three fresh fish on a platter) Clark: (points at one of them) That one.
c. Owner: Which fish do you want for dinner? Clark: (points at one of them) That one.

Note that whereas meaningful utterances of just about any modality can be clarified using a construction such as ‘what do you mean ‘…” (28a,b), clarification via repetition is limited to speech (28c,d). The first fact indicates that such utterances are viewed as having a content on a par with linguistic speech; the second fact demonstrates that clarification via repetition is not ‘anything goes’ and involves a construction that has clearly defined selectional restrictions (in contrast with certain other quotative constructions discussed in section 3.4):^10^

(28) a. A : (laughs). B: What do you mean he he? / Why are you laughing?
b. P1: my spine's like (*gestures spine shape*) (1.0 sec) like that (0.9 sec)
  P2: like this? (*gestures to clarify spine shape, quizzical face*) (From the corpus described in Healey et al. (2015))
c. A : (laughs). B: (laughs) [cannot mean ‘what’s the meaning of your laughter?]
d. A: (makes strange gesture) B: (repeats A's gesture) [cannot mean ‘what’s the meaning of this gesture you just made?].

Finally, we note two interactions between phenomena described earlier: first, the possibility of quoting gesture, as in (29):

(29) Claire: How pleasant, mum's being sick everywhere.I said erm is there a problem? (laugh) (vomiting noise) No. Not a problem. (BNC,KPH).

Second, the finding of Cook et al. (2009) that when speakers produce structures that they themselves usually disprefer, they are more likely to produce OCM utterances *and* to produce gestures.

## 4. Evidence from other disciplines

### 4.1. Language acquisition

Language acquisition has often been presented as the raison d'être of (formal) grammar. Since the mid 1960s Universal Grammar was proposed as a means of characterizing the knowledge children have as they acquire language and of course the ‘end state’ when the language is acquired (Chomsky, 1965; Snyder, 2007)^11^. In this paper we argue for a richer notion of grammar, but paradoxically this enriched notion is, we believe, a more promising theoretical notion as far as language acquisition/development is concerned than its interaction–free counterpart.

To what extent is interaction a necessary feature of language acquisition?^12^ In the extreme cases it is known that wolf children and children held in isolation do not acquire language in any normal sense (Lane, 1979; Curtiss, 2014). A far more difficult set of issues revolve around the fact that in a variety of cultures—e.g., Warlpiri and Mayan (Bavin, 1992; Brown, 2001)—infants are not considered potential or appropriate conversational partners, and so infants are not directly addressed by adults. And yet, language is acquired. Lieven (1994) argues that in such cultures language acquisition involves a significantly distinct trajectory. Nonetheless, despite anecdotal evidence suggesting slower development in such societies, there are various difficulties to compare rates of comprehension between the two types of developmental environments given different access to conversation for children. On the other hand, there is extensive evidence about differences in amount and type of utterances children are exposed to across distinct social socioeconomic status (SES). Most famously, Hart and Risley (1995) reported a ratio of approximately 4 : 2 : 1 for the total words heard by, respectively, American children of high SES parents middle SES parents, and lower SES parents. This is strongly correlated with speed of acquisition: by 3 years of age, the mean cumulative recorded vocabulary for the higher SES children was over 1000 words and for the lower SES children it was somewhat less than 500, whereas other studies show similar large effects on grammatical development (e.g., Snow, 1999).

To this one can add important experimental and corpus-based work on the efficacy and ubiquity of error correcting interaction between parents and children. In a series of papers using a paradigm of teaching nonsense verbs to young children, Saxton et al. (Saxton et al., 1998; Saxton, 2000) show that (i) learning on the basis of positive *and* negative evidence was significantly faster than learning solely on the basis of positive evidence; (ii) negative evidence has a long-term impact on the grammaticality of child speech. On a larger scale, Chouinard and Clark (2003) show, based on a detailed longitudinal study of 5 English and French speaking children, that negative evidence is supplied to a high percentage of children's erroneous utterances at all levels (phonological through syntactic).

An interaction–free view of grammar has to remain silent about such findings; approaches which view grammar as characterizing talk in interaction can correlate the quality of the interaction with speed and quality of intermediate states. Indeed, the repair notions we suggest belong in the grammar can, at least in principle, offer a basis for how interaction enables grammar modification to take place. We hasten to add that these findings have not yet been tied into formal models of learning (see e.g., Clark and Lappin, 2010). But this reflects the current state of the art in this field. Broadly speaking, there are currently two main approaches to the acquisition of grammar. There is nativism, inspired by Chomskyan assumptions (Chomsky, 1965; Snyder, 2007) and there is the usage–based approach (Tomasello, 2003). These two approaches differ radically on a number of dimensions: the nativist approach assumes the autonomy of syntax, whereas the usage–based approach takes constructions, conventionalized form-function units, as basic; for nativism the role of learning is limited to words and how these relate to Universal Grammar, whereas the usage-based approach highlights the importance of domain-general learning mechanisms such as analogy, entrenchment, and automatization. As things stand, however, neither nativist, nor the usage-based approach has advanced an explicit theory that would enable one to make clear predictions about how the grammatical system of a child evolves at various points as a result of conversational interaction with her carers or as an observer of such conversations. This is, in part, because, with very few exceptions (Ginzburg and Moradlou, 2013; Jackendoff and Wittenberg, 2014), the early stages of linguistic competence have not been formally described, presumably because of the significant challenge they pose for existing grammar frameworks.

### 4.2. Cognitive neuroscience

Earlier claims regarding e.g., the role of Broca's area in the processing of transformations notwithstanding (see e.g., Bambini, 2012 for a general survey of the neuroscience of language, and Grodzinsky, 2003 for an hypothesis about transformations in the brain), there is still a substantial disconnect between the research programs of cognitive neuroscience and theoretical linguistics, and the hypotheses that get formulated in those camps (Poeppel and Embick, 2005; Grimaldi, 2012). The primary interest of neurolinguists, cognitive neuroscientists studying language, is to identify the areas involved in different aspects of language processing; and there is now converging evidence that several areas are involved, above all the frontal lobe (e.g., Brodmann areas 44–Broca's area–45, 46, and 47), the temporal lobe (e.g., the superior temporal lobe, STL—Wernicke's area—and the superior temporal gyrus, STG), and parietal lobe (e.g., the angular gyrus; Bambini, 2012; Grimaldi, 2012). Such evidence clearly does not support either the claim of a separate 'faculty of language,' or the existence of a division between competence and performance (Grimaldi, 2012).

Some of the aspects of language use that we are proposing are governed by grammar, in particular turn-taking, have been studied in the field of **neuropragmatics** (Van Berkum, 2010; Bambini, 2012) but such studies show that the areas involved in such processing are the same, or very closely related, to those involved in aspects of language interpretation more traditionally accepted as involving competence. In fact, such studies tend to show that involvement in those aspects of language use results in greater activation of some of the areas associated with language processing. For instance, Jiang et al. (2012) found, using functional Near-Infrared Spectroscopy (fNIRS), that face-to-face dialogue results in increased activation in the inferior frontal cortex^13^ in comparison with back-to-back communication, or back-to-back monolog. And the comparison with face-to-face monolog strongly suggests that the difference in activation is primarily based on turn taking and body language.

Evidence concerning the *timing* of these interpretive processes doesn't clearly support their isolation from conventional aspects of grammatical interpretation either. Evidence by, e.g., Egorova et al. (2014) suggests that speech act identification and interpretation takes place rapidly—in fact, more rapidly than certain types of lexical-semantic processing.

## 5. Grammar for interaction : principles and illustration

One of the reasons for the relative neglect by linguists of phenomena such as those discussed in Section 3 is the apparent lack of adequate formal tools to describe their grammar. One of the key contentions of this paper, however, is that this is no longer the case, and that several frameworks for describing conversational contexts now exist which provide the tools to characterize the grammar of such phenomena. We then informally discuss how grammatical frameworks satisfying these constraints have been used to provide an account for the variety of phenomena discussed in Section 3. Our discussion will be sketchy and fairly informal, but in virtually all cases detailed, formally worked out treatments already exist to which references are provided.

### 5.1. Key theoretical assumptions

The interactionist view of grammar involves at least the following assumptions.

1. **Interaction situation reference** Grammars make essential reference to a dynamically updated **interaction situation** which indicates what is happening as the interaction takes place, along with some record of what has happened already.
2. **Sign instantiation** The grammar makes essential reference to certain **audio-visual-gestural events** that occur in the interaction event: uttering sounds, pointing, gesturing, etc.
  - **Incremental classification**: such events are classified into grammar-relevant types (**signs**) in incremental fashion by conversational participants.
  - **Partiality**: The classification process can be partial, where the type does not uniquely classify the event, thereby triggering repair.
  - **Non-monotonicity**: The classification process can be non-monotonic: the type assigned to an event can change as a consequence of repair.
3. **Event types in grammar rules** Linguistic generalizations and procedures are expressed not solely in terms of the events themselves but also in terms of *types* of events (or situations).
4. **Event type inference** Event types are used in rules which specify the enrichment of the interaction event by propositional and erotetic inference^14^.
5. **Language in flux** The class of grammatical types can be modified during interaction^15^.

**Interaction situation reference** is relatively uncontroversial: any grammar that treats indexicals like ‘I,’ ‘you,’ and ‘now’ needs to somehow effect reference to the interaction situation. However, the orthodox treatment (following Kaplan, 1978) is for this reference to be viewed as external to the grammar, formulated as indices relativizing the evaluation of sentences; the extent of indexicality assumed here and its explicitness yields significant novelty. By contrast, **language in flux** is operative in no major approach. It is however a key assumption for both language acquisition and repair.

The key innovations here are the assumptions we called **Event types in grammar rules**, **Event type inference**, and **Sign instantiation**. The latter has several components, which are pairwise independent (so a grammatical framework might satisfy one without satisfying one of the others). As we will see, these assumptions have several controversial consequences for a more traditional view of grammar.

For concreteness we will assume a particular specification of the interaction situation that developed in the dialogue semantic framework kos (Ginzburg, 2012), though there are a variety of alternative theories of this notion, from the original formulation in Barwise and Perry (1983) to ptt (Poesio and Rieser, 2010, 2011)^16^. It is important to emphasize that on the approach developed in both KoS and PTT, there is actually no single context or interaction situation. Rather, analysis is formulated at a level of information states, one per conversational participant. Each information state consists of two parts, a private part and the dialogue gameboard, inspired by Lewis (1979), that represents information that arises from public interactions. The structure of the dialogue gameboard (DGB) is given in Table 2. The *Spkr* and *Addr* fields allow one to track turn ownership; *Facts* represents conversationally shared assumptions; *VisualSit* represents the dialogue participant's view of the visual situation and attended entities; *Pending* represents moves that are in the process of being grounded and *Moves* represents moves that have been grounded; *QUD* tracks the questions currently under discussion, though not simply questions *qua* semantic objects, but pairs of entities which we call *InfoStrucs*: a question and an antecedent sub-utterance^17^. This latter entity provides a partial specification of the focal (sub)utterance, and hence it is dubbed the *focus establishing constituent* (FEC)^18^ (cf. *parallel element* in higher order unification–based approaches to ellipsis resolution e.g., Gardent and Kohlhase (1997) and Vallduví (2016) relates the focus establishing constituent with a notion needed to capture *contrast*.

**Table 2:** **Dialogue Gameboard**.

**Component**	**Type**	
Spkr	Individual	*keeps track of Turn*
Addr	Individual	*ownership*
utt-time	Time	
Facts	Set(propositions)	*Shared assumptions*
VisualSit	Situation	*Visual scene*
Moves	List(Locutionary propositions)	*Grounded utterances*
QUD	Partially ordered	*Live*
	set(〈question, FEC〉)	*issues*
Pending	List(Locutionary propositions)	*Ungrounded utterances*

### 5.2. Sign instantiation and its consequences

One of the types of events that are recorded in the Interaction Situation according to the sign instantiation hypothesis are **utterances**. Specifically, we assume that as the result of utterances taking place, the Interaction Situation is dynamically updated by recording pairs

〈utterance event, utterance type〉

where an utterance type is the equivalent of a **sign** in sign-based grammars such as Head Driven Phrase Structure Grammar (HPSG, Pollard and Sag, 1994; Ginzburg and Sag, 2000; Sag et al., 2003), Categorial Grammar (see e.g., Calder et al., 1988; Moortgat, 1997), or in versions of Lexical Functional Grammar (see e.g., Muskens, 2001). A pair 〈*u, T*_*u*_〉 indicating the occurrence of an utterance event *u* of type *T*_*u*_ is called a **locutionary proposition**. For instance, suppose that A utters (30a). Then Pending is updated by recording the locutionary proposition in (30b), stating the occurrence of utterance event *u*_*bk*_ of type Say(A, Bo kowtowed?).

(30) a. A: Bo kowtowed?
b. 〈*u*_*bk*_, Say(A, Bo kowtowed?) 〉

In fact, in versions of Interaction Grammar like kos or ptt every sub-utterance of *u*_*bk*_ expressing a constituent of *u*_*bk*_ gets recorded as a separate locutionary proposition: e.g., the utterance event *u*_*kowtow*_ of uttering the word *kowtow*.

(31) a. A: Bo kowtowed?
b. 〈*u*_*Bo*_, Say(A, ‘Bo') 〉
c. 〈*u*_*kowtow*_, Say(A, ‘kowtow') 〉

We will assume in this paper two main types of verbal interaction events–**Say** and **Ask**–as well a few other non-verbal interaction events discussed below.

#### 5.2.1. Other repair

As we discussed in Section 3.3, the grammar makes available various constructions whose primary function is to request clarification about prior utterances. We discuss here two cases—for detailed formal analysis see Ginzburg and Sag (2000), Purver (2006), and Ginzburg (2012).

An analysis of sentential reprises such as (32) involves a construction which, via reference to the interaction event, builds a content in the following way: the maximally pending utterance serves as the proposition from which a question is formed, indicated here using the notation ?*p*—zero or more argument roles are queried, corresponding to referential elements that cannot be resolved in context:^19^

(32)  a.  A: Do you like Hrvati? B: Do I like what?
        b.  MaxPending utterance content for A: Ask(A,?like(B,h))
        c.  MaxPending utterance content for B: Ask(A,?like(B,x))
             (B cannot resolve *x*)^20^
        d.  Content         of       B's     clarification      question:
             ?x Ask(A,?like(B,x)).

For an utterance like (33a), we need to say more about the reasoning an interlocutor makes when posing a clarification question. We assume that after every utterance the addressee engages in monitoring the incoming utterance *u*0: if she thinks she understands it—she can classify *u*0 with a fully instantiated sign, she responds accordingly; if not, taking as input her partially instantiated locutionary proposition, she has a right to accommodate into the context one of a small number of questions concerning any sub-utterance of *u*0 (Ginzburg and Cooper, 2004; Purver, 2006; Ginzburg, 2012). Thus, for any sub-utterance *u*1, the grammar enables reference to the question ‘what did prev-spkr mean by u1’ constrained by segmental phonological parallelism with u1. In other words, we assume the existence of a construction whereby a phrase segmentally identical to a sub-utterance u1 of the previous utterance can express a question like (33b):

(33) a. A: Did Bo kowtow? B: kowtow?
b. ?*x*.*Mean*(*A, u*_*kowtow*_, *x*) (“what did A mean by uttering ‘kowtow’?”)

What price do we need to pay to develop an account like this one of (33a)? The main cost involves the context: via Sign instantiation and Event type inference we assume that interlocutors maintain highly structured representations of utterances to enable them to engage in clarification question accommodation. Specifically, representations which specify the morphosyntactic and meaning representation for each sub-utterance, given the fact that each sub-utterance down to the level of the word is potentially clarifiable (Poesio, 1995; Poesio and Muskens, 1997; Purver et al., 2001, 2016; Poesio and Rieser, 2010, 2011).

As far as the grammar goes, the cost is this: the ability to specify constructions which make reference to elements of QUD. This latter requirement is currently supported by much evidence (Ginzburg, 1994, 2012; Roberts, 1996, 2004).

#### 5.2.2. Quotation

Given the ubiquity of quotation in natural language, linguists need to explicate the mechanisms it employs. Indeed, as we suggested earlier, one is obligated to do so in a way that offers an answer to the question: why, rather than being a heterodox linguistic process, is in fact quotation so straightforward?

The short answer, we suggest, is that this is because quotation involves entities and mechanisms utilized ubiquitously during dialogue processing. In other words, sign instantiation.

How does this apply to quotation? Ginzburg and Cooper (2014) postulate that pure quotations denote signs and direct quotations denote locutionary propositions. We illustrate how this applies to direct quotation briefly.

Direct quotation involves providing a *demonstration* of a previous communicative act *u* (or in extreme cases a sound or gesture act imbued with communicative intent) (de Cornulier, 1978; Clark and Gerrig, 1990)^21^. What varies with context is how similar the demonstration is going to be to *u* (does the demonstration use the same language? does it filter away disfluencies? how close in terms of content is it to *u*?).

By representing a direct quotation in terms of *u* (the original act) and *T* (the type corresponding to the demonstration), we can specify the similarity required in context.

A predicate embedding a direct quotation like English ‘like,’ ‘go,’ or French ‘faire’ is then posited to select for a locutionary proposition (*u, T*) and to predicate of the content of *u*. Thus, in (34a), A makes an utterance in French including the hesitation marker ‘euh’. B reports this utterance in English using the utterance ‘No way I'll do it’ which has filtered away the hesitation and whose content B views to be sufficiently similar to *u*_*A*_, A's utterance

(34)  a.  Je le ferai       euh genre absolument pas.
              I   it do-future uh  like    absolutely   not.
              ‘ I’ll do it uh like no way’
         b.  content(*u*_*A*_) = Not(Do(A,d))
         c.  B: He went ‘like no way I’ll do it’.
         d.  B’s        direct         quotation      of      A:          Say(B,
                Say(A,(u_*A*_,T_*‘no way I'll do it′*_)).

#### 5.2.3. Own communication management

Dealing with OCM does not require much change as far as context goes: the monitoring and update/clarification cycle is modified to happen at the end of each word utterance event—or in principle more frequently Brennan and Schober (2001)—, and in case of the need for change, a clarification question gets accommodated into QUD. Overt examples for such accommodation is exemplified in (35).

(35) a. Carol: Well it's (pause) it's (pause) er (pause) what's his name? Bernard Matthews' turkey roast. (BNC, KBJ)
b. A: Here we are in this place, what's its name? Australia.

The answer to this question is then used as the alteration and this triggers an update of the representation of the utterance (Ginzburg et al., 2014).

While the contextual background to OCM requires little change to the view of context outlined previously, accounting for OCM requires considerable changes in the outlook of the grammar. Specifically, it requires

1. an incremental and non-monotonic view of utterance construction.
2. ‘non-grammatical’ speech events to be incorporated within the domain of the grammar.

This latter assumption is required since words and collocations that constitute ‘editing phrases’ (e.g., ‘No’, ‘Or’, ‘I mean’) select for utterance events which can contain ‘ungrammatical’ aspects.

Hence, the status of the grammar shifts radically, potentially in line with views that argue for intrinsic gradience (Lau et al., 2016)^22^. It now characterizes as ‘well formed’ speech events that contain ill formed parts, albeit ones that have been corrected, for instance the German/Hebrew ones in (36a,c); a native speaker can distinguish these from potential utterances such as (36b,e,f) with no corrections or where the correction has gone awry:

(36)  a.   der          der           die         Batterie         die  versorgt  nur   im  Notfall.
              art-masc  art-masc  art-fem  Battery-fem   it    powers   only  in   case-of-need.
              ‘the the the battery it powers only in case of need’ (example (20), Fox et al. (2010))
         b.  ^*^die       die         der           Batterie        die versorgt  nur    im  Notfall.
              art-fem  art-fem  art-masc  Battery-fem  it    powers   only  in   case-of-need.
              ‘the the the battery it powers only in case of need’
         c.  kaxa she              hi    amda  he’emida            oto  leyad ha-luax.
              So    compl-decl  she  stood  stood-causative  it    near   def-blackboard.
             ‘In such a way that she stood placed it near the blackboard’ (example (26), Fox et al. (2010))
         d.  ^*^ kaxa she              hi    amda  oto  leyad  ha-luax.
              So       compl-decl  she  stood  it     near   def-blackboard.
              ‘In such a way that she stood it near the blackboard’
         e.  ^*^ kaxa she              hi    he’emida            amda  oto  leyad  ha-luax.
              So       compl-decl  she  stood-causative  stood  it    near   def-blackboard.
              ‘In such a way that she placed stood it near the blackboard’.

#### 5.2.4. Interjections and turn assignment

Consider a word like ‘marḥabteyn.’ As we discussed in Section 3.1, this word is used as a response greeting by Bilal just in case the initial greeting by Awda was ‘marḥaba.’

In a grammar which enables reference to the interaction event, this is easy to capture: such a word has a presupposition about the form and content of the previously grounded move, that its form was ‘marḥaba’ and content a greeting.

What of turn assignment utterances, as in (16)? As with greetings, in a grammar that allows reference to the interaction event, which tracks turn holders, an utterance which expresses a wish about a projected turn holder is easy to encode.

### 5.3. Non-sentential utterances

In Section 3.1, we pointed out that the content one assigns to a non-sentential utterance like ‘four croissants’ can vary widely, with the sources for the different contents ranging from a previously uttered question through domain–specificity and to a correction. We have also emphasized that different non-sentential utterance constructions exhibit morphosyntactic and/or phonological parallelism with their antecedents, which in the case of short answers can be maintained across multiple turns. This means that not only does the combinatorial semantics of non-sentential utterance constructions integrate information from the Dialogue GameBoard, but that this is also potentially true of the morphosyntactic and phonological specifications of such constructions. Such information needs to be projected into the context, as we have already observed in the case of repair constructions, maintained, in parallel with QUD–oriented information.

We claim that it is only with a theory of interaction that structures the context appropriately that we can capture the uniformity underlying such utterances. We do so via a construction type, as in (37e) which generalizes a rule proposed already in Hausser and Zaefferer (1979). Its content field involves the following predication: the predicate is the question under discussion, whereas the subject is the bare non-sentential utterance; the rule's syntactic specification requires that the non-sentential utterance bears the same syntactic category as its antecedent in QUD, the focus establishing constituent (fec):

(37) a. B: Four croissants.
b. (Context: A: What did you buy in the bakery?) Content: I bought four croissants in the bakery.
c. (Context: A: (smiles at B, who has become the next customer to be served at the bakery.)). Content: I would like to buy four croissants.
d. (Context: A: Dad bought four crescents.) Content: You mean that Dad bought four croissants.
e. *Declarative-fragment-clause*:
  Cont = DGB.MaxQUD(u_*nsu*_.cont)
  u_*nsu*_.cat = MaxQUD.fec.cat : Syncat.

For a detailed analysis of a wide range of NSU constructions found in the BNC see Fernández (2006) and Ginzburg (2012).

What of cases such as (27), repeated here as (38)? The answers get introduced into the semantics via mechanisms discussed in Lücking et al. (2015), whereas the question via domain specific (or alternatively *genre–based*) inference (Larsson, 2002; Ginzburg, 2012)^23^.

(38) a. Owner: (displays three fresh fish on a platter) Clark: (points at one of them) (From Clark (2012): example (32))
b. Owner: (displays three fresh fish on a platter) Clark: (points at one of them) That one.

### 5.4. Order-dependent expressions

One of the key theoretical assumptions listed in Section 5.1 is that the Interaction Situation includes a locutionary proposition for every single word. Using this assumption we can provide an exhaustive account of order-dependent expressions.

Using the notation introduced above 〈*u*,Φ〉 to state that utterance event *u* is of type Φ, and assuming a function ↝ mapping utterance events to their content, we can say that the result of uttering the NP *Bob* is to update the current utterance (the maximal element of Pending) by adding to it the two conditions in (39). The first one records the utterance *u* by A of the word-string “Bob”; the second one records that the content of the utterance event *e* is the object denoted by *b*. (We assume here a ‘natural’ semantics for proper names as proposed by Partee, together with type raising operations.) Subsequent utterances of the expressions *and, John*, etc. update the common ground in a similar fashion, by adding new utterance events preceded by *e*.

(39) {〈*u*,**Say***(A,“Bob”)*〉, *u* ↝ *b* }

It should be easy to see how the framework just sketched can be used to specify the interpretation of order-dependent expressions like *the former one* and *vice versa*. The meaning of the two expressions can in fact be characterized as follows:

**the former one** The content of an event of uttering an NP of the form *the former N* is that element *x* of a set X of familiar objects which is also the content of the first utterance event *u*_1_ among those used to introduce the elements of X^24^.

The required constraints on the Interaction Situation are imposed by assigning to *former* the following interpretation. Say that *u* is an event of saying “former”:

(40) 〈*u*,**Say***(A,“former”)*〉

The interpretation of *u* consists a 'linguistic' part (the content of *u*) and a ‘metalinguistic’ one. This second part imposes conditions on the Interaction Situation: namely, the requirement that two events of uttering nominals *u*_1_ and *u*_2_ occurred in the interaction event, *u*_1_ preceding *u*_2_ and having content *x*. The first part then specifies that the content of the utterance of the adjective *former* is a predicate modifier specifying the restriction that the object to which the predicate is applied must be equal to *x*.

**vice versa** The content of an event of uttering the string *vice versa* conjoined with an utterance with contextually specified constituents u_1_ … u_*n*_ is obtained by applying the usual rules of semantic interpretation to combine the contents of u_1_ … u_*n*_, after having switched two contextually specified utterance events u_*i*_ and u_*j*_ that are part of u_1_ … u_*n*_. For instance, in the case of (15b) in Section 3.2.3, *I think actors can teach dancers a lot, and vice versa*, the content of the event of uttering *vice versa*, which is conjoined with the contextually specified sequence of events u_1_ … u_6_ of uttering *actors*_1_ *can*_2_ *teach*_3_ *dancers*_4_ *a*_5_ *lot*_6_, is obtained by applying the usual rules of semantic composition to the sequence obtained by switching u_1_, *actors*, with u_4_, *dancers*.

### 5.5. Anaphora

We have shown in, e.g., Poesio and Rieser (2010) and Poesio and Rieser (2011), that by adopting an interactionist approach to grammar the examples discussed in Section 3.2.1 can be analyzed within a treatment of anaphora that is a natural extension of Discourse Representation Theory (Kamp and Reyle, 1993) and is closely related to, e.g., the proposals in Asher and Lascarides (2003). In such extensions, updating the Interaction Situation with new locutionary or illocutionary events makes new discourse referents available just as events are in the situation under discussion. As a result, implicit anaphoric references such as those in (41a), repeated here for convenience, can be handled precisely as shown in (41b), which specifies the occurrence in the interaction situation of two speech acts *ce*1 and *ce*2 (**conversational events** in ptt). These two speech acts are related by a **concession** rhetorical relation.

(41) a. Although MSG [Monosodium Glutamate] has been blamed for a variety of symptoms, it has been vindicated by scientific research.
b. 〈*ce1*, **assert***(writer, ‘MSG has been blamed for a variety of symptoms”)*〉,
  〈*ce2*, **assert***(writer, ‘MSG has been vindicated by scientific research’)*〉,
  **concession**(*ce1*,*ce2*)

Within this framework, *explicit* references to illocutionary acts as in (42b), where *that* is a reference to the (speech) act of promising in (42a), can be handled similarly as the implicit references to such events found in SDRT:

(42) a. A: John, I promise I will help you with your homework.
  〈*ce1*, **promise***(A,‘A will help John with his homework’)*〉
b. B: That was silly, as you won't have any time.
  〈*ce2*, **assert***(B,‘ce1 was silly as A won't have any time’)*〉

The references to locutionary events as in the example from Webber (1991) ‘4’ can be analyzed in a similar way provided we assume that not only illocutionary events, but locutionary events as well, are part of the interaction event:

(43) a. A: The combination is 1-2-3-4.
b. 〈*u1*, **Say***(A,“the combination is 1-2-3-4”)*〉
c. B: Could you repeat that? I didn't hear it.
d. 〈*u2*, **Say***(B,“could you repeat u1? I didn't hear u1')*〉

### 5.6. Pointing and gestures

A grammatical framework in which grammar imposes constraints on the Interaction Situation is naturally suited to specify grammatical constraints on other aspects of communication such as gestures and pointing, as these are just other types of events whose occurrence is recorded in the Interaction Situation. In Rieser and Poesio (2009) we proposed that propositions of the form

〈g,G(A)〉

where *G* is a type of gesture, are recorded in the Interaction Situation to indicate the performance of a grammatically relevant type of gesture by A. An example of grammatically relevant gesture is pointing:

〈p,point(A)〉

(from which we can indirectly infer, following the type of reasoning studied by Lücking et al., that

〈p,point-at(A,ϕ,)〉

A multimodal grammar for the integration of pointing and speech based on this treatment of gestures in the Interaction Situation was proposed in Poesio and Rieser (2009). Clearly, the framework could also be used to provide an account of gestures referring to other aspects of the Interaction Event–e.g., for turn-taking.

## 6. Discussion

### 6.1. The initial data revisited: contextualizing compositionality

We started the paper by using two real dialogues to illustrate the challenges that interaction poses for contemporary grammars. In Section 5 we then proposed a number of principles that enable grammars to analyze spoken language and sketched accounts of various phenomena introduced in Section 3. To what extent do these help with the initial dialogues from Section 1?

#### 6.1.1. Disfluencies

Our preferred terminology is *own management communication*, which emphasizes the intentional and useful nature of such phenomena. We provided an example of the type of approach that explicates their coherence and situates them within the ubiquitous aspects of utterance processing.

#### 6.1.2. Non sentential utterances and interjections

Again, we provided a basic approach here, with references to highly detailed, formalized accounts elsewhere. The example account we provide involves constructional/lexical specifications that can interface directly with dialogue context that contains both linguistic and non-linguistic information.

#### 6.1.3. Overlapping turns

We argue that a key desideratum with respect to turn management is incremental classification of speech events, as in the example account provided. This account also emphasizes that each conversationalist has their own view of the interaction situation—the dialogue gameboard. These are important ingredients in tackling this phenomenon, that will have to be provided in a proper account.

#### 6.1.4. *Ad hoc* coinages

We have emphasized as a key principle that grammars are open and non-global. This is crucial for acquisition, repair, and quotation. We have scratched the surface with respect to this in our discussion of the latter two.

#### 6.1.5. Compositionality

In Section 1 we argue that the grammars need to encode a view of compositionality whereby meaning emerges by combining information from the interaction situation, speech events, and gestures. One very simple example of such a notion—*sans* gestures—is given in our rule for declarative fragments given in (37) in which both meaning composition and morphosyntactic parallelism are driven by the dialogue gameboard. For rules integrating gesture in a similar framework, see e.g., Lücking (2016).

### 6.2. Moving the boundary between competence and performance

Let us assume, initially, for argument's sake that a competence/performance distinction is tenable as the basis for a theory of the human language faculty. What we have shown in this paper is that the boundary as commonly drawn is entirely artificial as it leaves out a host of key aspects of interaction that are clearly governed by ‘grammar’ under any sensible notion of what a ‘grammar’ is.

A secondary but still key aspect of our proposal is that this redrawing of the boundary does not in any way involve abandoning the aim of providing a formal account of the structure and meaning of language in interaction. To be sure, there is still a lot of work to be done in developing a formal ‘Interaction Grammar’ framework that may provide as productive a foundation for theories of the extended notion of grammatical competence as the ‘standard theory’ that emerged in the 1970s and 1980s from the work of Chomsky, Montague, Partee, Bresnan, Sag, and many others. But we believe that for all its necessary sketchiness the proposal in Section 5 shows what the essential ingredients of such a formalism would be; much more detailed developments have appeared in e.g., Ginzburg (2012).

There are two concrete results we can point to. First, we have demonstrated (building, in part, on insights that have been around for many years, but have repeatedly been forgotten) that the disembodied, context independent notion of grammaticality still much discussed (see e.g., Gibson and Fedorenko, 2013; Sprouse and Almeida, 2013; Lau et al., 2016) and which serves as one of the main empirical evaluation criteria for formal grammars is untenable and must be replaced by a contextually relativized notion. Second, the accounts we sketch for various of the phenomena at issue (interjections which presuppose prior use of other interjections, non-sentential utterances which carry structural presuppositions, self-repair, quotation) show how such a notion can be constructed.

The approach we propose here also displays what we hold to be a key property of any future framework of this type: i.e., that it doesn't overly muddy the grammatical baby with the interactional bathwater, i.e., that it is an extension and a generalization of the frameworks currently in use so that it does not require rethinking current grammar theory wholesale, as for many phenomena there already exist satisfactory accounts. Also, such an extension and generalization would allow linguists interested in phenomena that do not appear to involve reference to the interaction event to use only the formal machinery that is strictly required.

A third contention we have tried to exemplify throughout is that redrawing the boundaries this way will make work on grammar by theoretical linguists much more relevant to sister disciplines such as computational linguistics, conversation analysis, corpus linguistics, psycholinguistics, speech processing, the study of multimodal interaction, or cognitive neuroscience that in recent years have had to develop their own foundational frameworks as the formal tools provided by theoretical linguistics were too limited (Ferreira, 2005; Poeppel and Embick, 2005; Steedman, 2013).

### 6.3. The grammar-pragmatics boundary

We expect several readers of this paper will react by saying ‘interesting phenomena, but this is not grammar, it's pragmatics.’ Charting the semantics/pragmatics boundary is not easy (for some recent discussions, see Recanati, 2010; Stojanovic, 2013; Lepore and Stone, 2014 and there are certainly influential proposals suggesting that pragmatics intrudes in various incontrovertibly grammatical processes Levinson, 2000; Ariel, 2008). Avoiding these difficult issues here, we note that of the five classes of phenomena discussed, Grammar across turns, Online repair, Genre dependent grammar, Speech-gesture integration are all concerned incontrovertibly with structural issues or issues of meaning composition. This leaves the class of phenomena concerned with reference to the interaction event: we pointed out that other communication management constitutes the primary/literal meaning of a number of words and constructions, hence integrating these in grammar is as justified as integrating tense, which involves ordering relations between a described event and an utterance event (in our terminology—the interaction event.).

### 6.4. The place of the sentence in a theory of grammar

In traditional grammar, the notion of ‘sentence’ plays a central role; indeed, in formal grammars, a grammar is usually defined as a set of formal rules characterizing the sentences of the language. An important consequence of the adoption of an interactionist view is that this centrality needs to be reconsidered. In real conversations complete sentences without repair are far from being the rule; moreover, non-sentential utterances of various kinds are extremely common, as discussed in the previous sections.

### 6.5. Rethinking competence v. performance as black box v. white box testing

The competence/performance distinction is *prima facie* attractive because it enables one to separate analysis of ‘the linguistic phenomena’ from the specific details of how they get processed. The problem, we think, is that this reasonable desideratum has lead to a highly selective and misleading view of what are the ‘rule governed’ phenomena associated with language. We think a better construal of this separation could be drawn from computer science, which offers the distinction between black box and white box testing (Patton, 2006): the former pertains to the functionality of an application without peering into its internal structures or workings, whereas the latter involves trying to assess functionality, in part, by examining the implemented code.

## 7. Conclusions

In this paper we have presented compelling evidence to suggest that the view of grammar thus far predominant in formal linguistics, which relegates a variety of conversational phenomena to performance rather than grammatical competence, results in a overly impoverished view of our knowledge of language. We have argued for the need for a notion of grammaticality relativized to interaction situations. This, in turn, requires grammatical knowledge to be conceptualized dialogically, i.e., embedded within conversational interaction. We have also suggested that extending our view of grammar does not amount to a jump into the unknown: a number of frameworks are already emerging supplying us with the formal tools required to provide accounts of many such phenomena. Finally, we suggested that while no unified ‘Interaction Grammar’ yet exists, a few common assumptions among these frameworks can already be identified, which may lead to the development of such a theory.

## Author contributions

JG and MP conceived the paper, drafted the paper, and gave final approval for its publication.

## Funding

JG acknowledges support by the French Investissements d'Avenir–Labex EFL program (ANR-10-LABX-0083) and by the Disfluences, Exclamations, and Laughter in Dialogue (DUEL) project within the Projets Franco-Allemand en sciences humaines et sociales of the Agence Nationale de Recherche (ANR) and the Deutsche ForschungGemeinschaft (DFG), and by a senior member fellowship from the Institut Universaitaire de France, and, for its inspiring atmosphere, the café in the Israel Museum, Jerusalem. MP acknowledges support from the European Research Council project Disagreements in Language Interpretation (DALI), ERC-2015-AdG; and from the ESRC-funded Human Rights, Big Data and Technology project.

### Conflict of interest statement

The authors declare that the research was conducted in the absence of any commercial or financial relationships that could be construed as a potential conflict of interest.

## Footnotes

^1^Frazier (2014) proposes a somewhat related strategy whereby in addition to the standard competence grammar, there exists a repair system which functions as an automatic speech error reversal system where the repaired meaning is plausible and fits with presumed intent of the speaker.

^2^Of course, as an anonymous reviewer reminds us, this applies with equal force to scholars who do not accept the competence/performance distinction, who would presumably not dispute the importance of developing a grammar for such phenomena.

^3^This summary is based on Kendon (2004), chapters 3–5.

^4^‘Hello’ has an ironic use (‘Are you at all plugged in?’) that is not conversation–initial, a use that has also become conventionalized, as indicated by the fact that ‘Hi’ lacks this use.

^5^A similar datum from Estonian is pointed out to us by an anonymous reviewer for Frontiers: with the phrase ‘jōudu tarvis’ ([sufficient] strength [will be] necessary) requiring as preceding utterance the phrase jōudu' (‘'[may you the necessary] strength’). This latter is typically uttered on passing anyone working on the street/in their garden etc.

^6^For additional data about such cross-turn presuppositions see Linell (2009) and Linell and Mertzlufft (2014) on the Swedish and German *x-och/und-x* and initial double auxiliary constructions.

^7^The term ‘own communication management’ is due to Jens Allwood, see e.g., Allwood et al. (2005) for discussion.

^8^See also data from http://itre.cis.upenn.edu/~myl/languagelog/archives/003011.html and from Yuan et al. (2006), showing the regularity of the *extent* of speech events.

^9^We owe this datum to an anonymous reviewer for Frontiers.

^10^With respect to (28d), it's unclear whether repetition of a gesture accompanied by a quizzical face conveys a clarification request; Catherine Pelachaud (p.c.) has suggested to us that it might; this is currently the subject of an experimental study. However, at least in corpora where gesture clarification has been studied, one apparently finds only examples like (28b), as in the corpus described in Healey et al. (2015), data we thank Nicola Plant (p.c.) for.

^11^We use scare quotes for ‘end state’ because changes in adult grammar as a result of repair phenomena we have detailed above are a key feature of the notion of grammar we advocate.

^12^For a much more detailed discussion than we can offer here, on which we draw extensively, see Hoff (2006).

^13^Specifically, they seem to refer to Broca's area–see Jiang et al. (2012), Figure 1.

^14^That is, inference whose conclusion is, respectively, a proposition or a question—we exemplify both kinds of inference below.

^15^We borrow this term, originally due to Ruth Kempson, from Cooper and Ranta (2008) and Cooper (2012), who argue for a view of natural language grammar as a collection of resources that a linguistic agent has available in order to build local languages on the fly.

^16^Neither kos nor pttis an acronym.

^17^Extensive motivation for this view of QUD can be found in Fernández (2006) and Ginzburg (2012), based primarily on semantic and syntactic paralleism in non-sentential utterances such as short answers, sluicing, and various other non-sentential utterances.

^18^Thus, the focus establishing constituent in the QUD associated with a *wh*-query will be the *wh*-phrase utterance, the focus establishing constituent in the QUD emerging from a quantificational utterance will be the NP utterance, whereas the focus establishing constituent in a QUD accommodated in a clarification context will be the sub-utterance under clarification

^19^In the limit, no roles are queried and the question is a polar one, posed to confirm the intended content:

- A: Do you like Hrvati? B: Do I like Hrvati?
- MaxPending utterance content for A: Ask(A,?like(B,h))
- Content of B's clarification question: ?Ask(A,?like(B,h)) (“Are you asking if I like Hrvati”)

^20^Here ‘x’ denotes a contextual parameter that B cannot be resolved. For a technically precise explication of such a notion see Ginzburg (2012).

^21^As Ray Jackendoff (p.c.) reminds us, this need not hold for negative (‘She never said ‘ …' ’) or hypothetical (‘If I ask ‘…' ’) direct quotations. This is an instance of a more general interaction between negation, conditionalization, and event reference.

^22^Also relevant in this respect are *pivot constructions* discussed in Norén and Linell (2013); frequent in conversation, neither self–, nor other–corrected, violating basic selectional principles:

(i) E: oh that's what I'd like to have is a fresh one. (Norén and Linell, 2013, example 1).

^23^The essential idea of these proposals is that a given domain/genre can be characterized, in part, by a partially ordered set of questions, discussion of which constitutes its defining subject matter. At appropriate points these questions can be deduced as relevant and accommodated into QUD without being uttered overtly. For instance, in a customer/client interaction, the issue ‘what does the client require’ can become QUD-maximal.

^24^The meanings of events of uttering *the latter N, the first N*, …*the last N, the former one*, etc. are specified in a similar way.
